# Perirenal Fat Thickness: A Surrogate Marker for Metabolic Syndrome in Chinese Newly Diagnosed Type 2 Diabetes

**DOI:** 10.3389/fendo.2022.850334

**Published:** 2022-03-16

**Authors:** Xiu Li Guo, Mei Tu, Yang Chen, Wei Wang

**Affiliations:** ^1^ Department of Radiology, Longyan First Affiliated Hospital of Fujian Medical University, Longyan, China; ^2^ Department of Endocrinology, Longyan First Affiliated Hospital of Fujian Medical University, Longyan, China

**Keywords:** perirenal fat thickness, metabolic syndrome, newly diagnosed type 2 diabetes, optimal cut-off value, visceral adipose tissue

## Abstract

**Objective:**

Increasing evidence suggested that perirenal fat thickness (PrFT) was associated with metabolic risk factors. This study aimed to assess the association between PrFT and metabolic syndrome (MetS) in Chinese newly diagnosed type 2 diabetes (T2DM), further evaluating the ability of PrFT in identifying MetS.

**Method:**

A total of 445 Chinese newly diagnosed T2DM were enrolled in this study from January to June 2021. Demographic and anthropometric information were collected. PrFT was evaluated by CT scan on Revolution VCT 256. MetS was based on the Chinese Diabetes Society definition. Receiver operating characteristic (ROC) curve was conducted to assess the optimal cutoff value of PrFT in identifying MetS.

**Results:**

Overall, the prevalence of MetS was 57.5% (95% CI: 54.0–64.0%) in men and 58.9% (95% CI: 52.3–65.5%) in women separately. The correlation analysis showed that PrFT was significantly correlated with metabolic risk factors like body mass index, waist circumference, triglycerides, high-density lipoprotein cholesterol, systolic blood pressure, diastolic blood pressure, uric acid, and insulin resistance. PrFT was also shown to be independently associated with MetS after adjustment for other confounders. The odds ratios (ORs, 95% CI) were 1.15 (1.03–1.38) in men and 1.31 (1.08–1.96) in women (*P <* 0.05). The ROC curves showed a good predictive value of PrFT for MetS. The areas under the curve of PrFT identifying MetS were 0.895 (95% CI: 0.852–0.939) in men and 0.910 (95% CI: 0.876–0.953) in women (*P <* 0.001). The optimal cutoff values of PrFT were 14.6 mm (sensitivity: 83.8%, specificity: 89.6%) for men and 13.1 mm (sensitivity: 87.6%, specificity: 91.1%) for women.

**Conclusions:**

PrFT was significantly associated with MetS and showed a powerful predictive value for it, which suggested that PrFT can be an applicable surrogate marker for MetS in Chinese newly diagnosed T2DM.

**Clinical Trial Registration:**

This study was registered in clinicaltrials.gov (ChiCTR2100052032).

## Introduction

Metabolic syndrome (MetS) is the common pathological basis and early stage of many major diseases that is characterized by the simultaneous presence of obesity, hypertension, dyslipidemia, and hyperglycemia in individuals, leading to increased prevalence of cardiovascular disease (CVD) and stroke and risk of diabetes (1). Type 2 diabetes (T2DM) is a kind of metabolic disease characterized by chronic hyperglycemia due to the failure of pancreatic islet β-cells to sustain the hyperinsulinemia required to compensate for insulin resistance and often accompanied with other metabolic disorders. The prevalence of MetS has rapidly increased in China. A cross-sectional survey reported the prevalence of MetS in diabetes which is in the range from 53 to 68.1% (2). Due to the great harm and high prevalence of MetS in newly diagnosed T2DM, early diagnosis is urgently needed, whereas the diagnosis and awareness rate are suboptimal in clinical diagnosis and treatment (3). The diagnostic process for MetS in patients with diabetes is cumbersome and thus may limit the early diagnosis of MetS (4). An effective surrogate marker for MetS can help clinicians in identifying MetS in newly diagnosed T2DM.

Visceral adipose tissue is considered to be a type of “ectopic fat” which has adverse influences on systemic inflammation, insulin resistance, and metabolic profiles and, finally, increases the risk of developing MetS and CVD (5–7). Among visceral adipose tissue deposits, perirenal fat is located around and enclosed from the inner side of the abdominal musculature to the surface of the kidney, which can be easily measured by ultrasound, CT, and MRI scanning (8). Anatomical studies demonstrated that perirenal fat may modulate the metabolism system through neural reflexes, adipokine secretion, and adipocyte interactions due to its unique structure compared with other connective tissues (9–11). Thus, these features may provide a basis for the involvement of perirenal fat in MetS regulation. Cross-sectional studies also observed that perirenal fat thickness (PrFT) is associated with the components of MetS, such as hypertension, obesity, and dyslipidemia (12, 13). Based on the above-mentioned anatomical and cross-sectional studies, it may indicate to us that PrFT can be a surrogate marker for MetS. Hence, we design a cross-sectional study to assess the association between PrFT and MetS in Chinese newly diagnosed T2DM, further evaluating the ability of PrFT in identifying MetS.

## Study Design and Methods

### Study Design and Participants

This cross-sectional study consecutively enrolled individuals from the Department of Endocrinology Clinic who were screened for diabetes at the Longyan First Affiliated Hospital of Fujian Medical University and who fulfilled the study criteria between January 2021 and June 2021. The study inclusion criteria were as follows: (1) newly diagnosed T2DM using the World Health Organization (WHO) 2019 criteria (fasting plasma glucose ≥126 mg/dl or 2-h postprandial ≥200 mg/dl during oral glucose tolerance test (OGTT) or HbA1C ≥6.5% or a patient with classic symptoms of hyperglycemia or hyperglycemic crisis or with random plasma glucose ≥200 mg/dl and (2) autoimmune antibodies like glutamic acid decarboxylase antibody (GADA), insulin autoantibody (IAA), and islet cell autoantibody (ICA) negative. Participants were excluded if they were any of the following cases: (1) pregnancy at diagnosis or gestational diabetes mellitus, (2) secondary or special type of diabetes, (3) presence of acute diseases that could interfere with glucose metabolism, (4) with renal structure abnormalities (tumors and cysts or history of renal region surgery), and (5) currently receiving lipid-lowering therapies. In this study, we estimated the sample size according to the requirement of multiple binomial logistic regression model; 10–12 variables may be put into the logistic regression model according to the principle of 5–10 events per variable (14), and the prevalence of MeTS is about 53 to 68.1% (2). Thus, we planned a sampling size of 400–500 patients. The definition of newly diagnosed T2DM is as follows: previous unknown hyperglycemia status and diagnosed with T2DM for the first time (15). Metabolic and hormonal parameters were assessed, and demographic and anthropometric information were evaluated. Then, a CT scan was performed on all participants to measure PrFT. All procedures were conducted in accordance with the Declaration of Helsinki. This study was approved by the Ethical Committee of Longyan First Affiliated Hospital of Fujian Medical University (LY-2020–069) and registered in clinicaltrials.gov (ChiCTR2100052032). All participants enrolled in the study provided informed consent.

### Definition of Metabolic Syndrome

MetS was defined according to the Chinese guideline for diabetes with MetS management (16). Patients who meet three or more of the following criteria are considered to have MetS: (1) abdominal obesity: waist circumference (WC) ≥90 cm in men or ≥85 cm in women, (2) hyperglycemia: fasting blood glucose (FBG) ≥6.1 mmol/L or (OGTT) 2-h blood glucose ≥7.8 mmol/L or previously diagnosed diabetes with treatment, (3) hypertension: blood pressure ≥130/85 mmHg or currently under anti-hypertension therapy, (4) fasting triglycerides (TGs) ≥1.70 mmol/L (without lipid-lowering therapies), (5) fasting high-density lipoprotein cholesterol (HDL-c) <1.04 mmol/L. All patients in this study fulfilled the criteria for hyperglycemia and were diagnosed as newly diagnosed T2DM.

### Anthropometric Measurements and Metabolic Parameters

Demographic information was collected through a standard questionnaire *via* face-to-face interviews by a physician (mainly including gender, age, history of medication, disease, surgery, family diabetes, and current or ever-smoking). Information was also obtained by a review of medical records and laboratory data. Physical examination was conducted by the research nurses (including height, weight, WC, and blood pressure). Body mass index (BMI) was calculated as the weight (kg, rounded to the nearest kilogram) divided by the square of height (m, rounded to the nearest centimeter). WC was measured at the anatomical waist (the natural depression between the iliac crest and the 10th rib), which should be the narrowest part of the abdomen. Systolic and diastolic blood pressure (SBP and DBP) were recorded on at least three different occasions by an electronic sphygmomanometer with an appropriate cuff size after the patients have rested for more than 5 min, and the three readings were calculated.

Serum FBG, insulin, glycosylated hemoglobin, autoimmune antibodies (GADA, IAA, and ICA), total cholesterol (TC), HDL-c, LDL-c, TGs, uric acid (UA), and high-sensitivity C-reactive protein were measured by standard methods using fasting venous blood samples that were taken between 8 and 9 a.m. after overnight fasting for at least 12 h. Homeostasis model assessment (HOMA-IR) was used to assess insulin resistance. The estimate of HOMA score was calculated with the formula: fasting serum insulin (µU/ml) fasting plasma glucose (mmol/l)/22.5 (17).

### Measurement of Perirenal Fat Thickness

**Figure 1:**
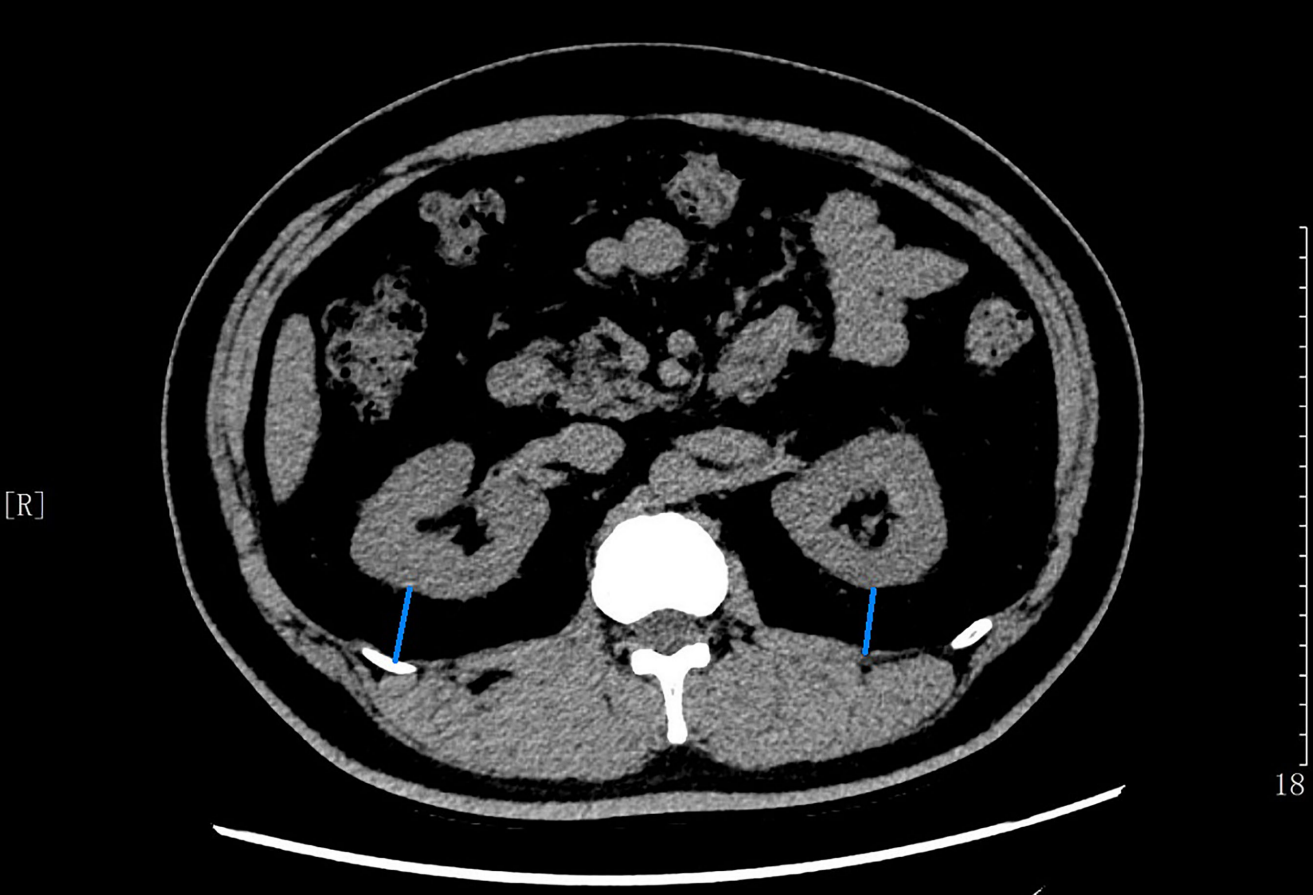
The average of maximal thickness values (blue line) between the posterior wall of the kidney and the inner limit of the abdominal wall across the renal venous plane was calculated as the PrFT.

CT scan was performed on all patients using Revolution VCT 256 (General Electric, Milwaukee, WI, USA) while in a supine position to measure PrFT. Images were reconstructed with Advantage work station 4.7 software (GE, Milwaukee, WI, USA) to obtain 1.25-mm-thick consecutive slices. The CT-scanned area covered was between the pubic symphysis and the 10th thoracic vertebra. Perirenal fat was differentiated from other tissues by density (HU). The center of the window is set in -100 HU, and the window widths ranged from 50 to 200 HU for further analysis. Each compartment is drawn by using a manually controlled trackball cursor. PrFT was measured on both sides for each patient according to a recent study (8). The adipose area of the renal sinus was separated by a tangent line touching the outer limits of the kidney and crossing over the renal hilum. Moreover, perirenal fat was separated by tracing the boundaries of the kidney, the aforementioned tangent line, and the perirenal fascia. The average of the maximal distance between the posterior wall of the kidney and the inner limit of the abdominal wall across the renal venous plane on both kidneys was calculated as the PrFT (**Figure 1**). Two radiologists were involved in the measurement of PrFT to reduce the inter-operator variability. The inter-operator agreement between the two radiologists is 0.92.

### Statistical Analysis

Data were analyzed by using SPSS 23.0 software (SPSS Inc., IBM). Descriptive data were expressed as means ± standard deviation (SD). Discrete variables were summarized in frequency tables (*N*, %). The patients enrolled in this study were divided into three groups based on the tertiles of PrFT. The Cohen k statistic was used to assess the agreement of the PrFT measurements between the two radiologists. Statistical differences among groups were established with one-way analysis of variance (ANOVA), followed by Tukey test for multiple comparisons. Chi-square (*χ* ^2^) test or Fisher’s exact test was used for the comparison of categorical variables. Student’s *t*-test was used to compare the mean PrFT between the two genders. The relationship between PrFT and the metabolic parameters was assessed using Pearson’s or Spearman’s correlation analysis. A multiple binomial logistic regression model was used to estimate the independent effect of PrFT on the MetS after adjusting for other covariates by gender. The ROC curve was used to evaluate the identifying value of PrFT for MetS in newly diagnosed T2DM. The optimal cutoff value was based on the greatest value of the Youden index. A two-tailed value of *P <*0.05 was considered statistically significant.

## Results

**Figure 2:**
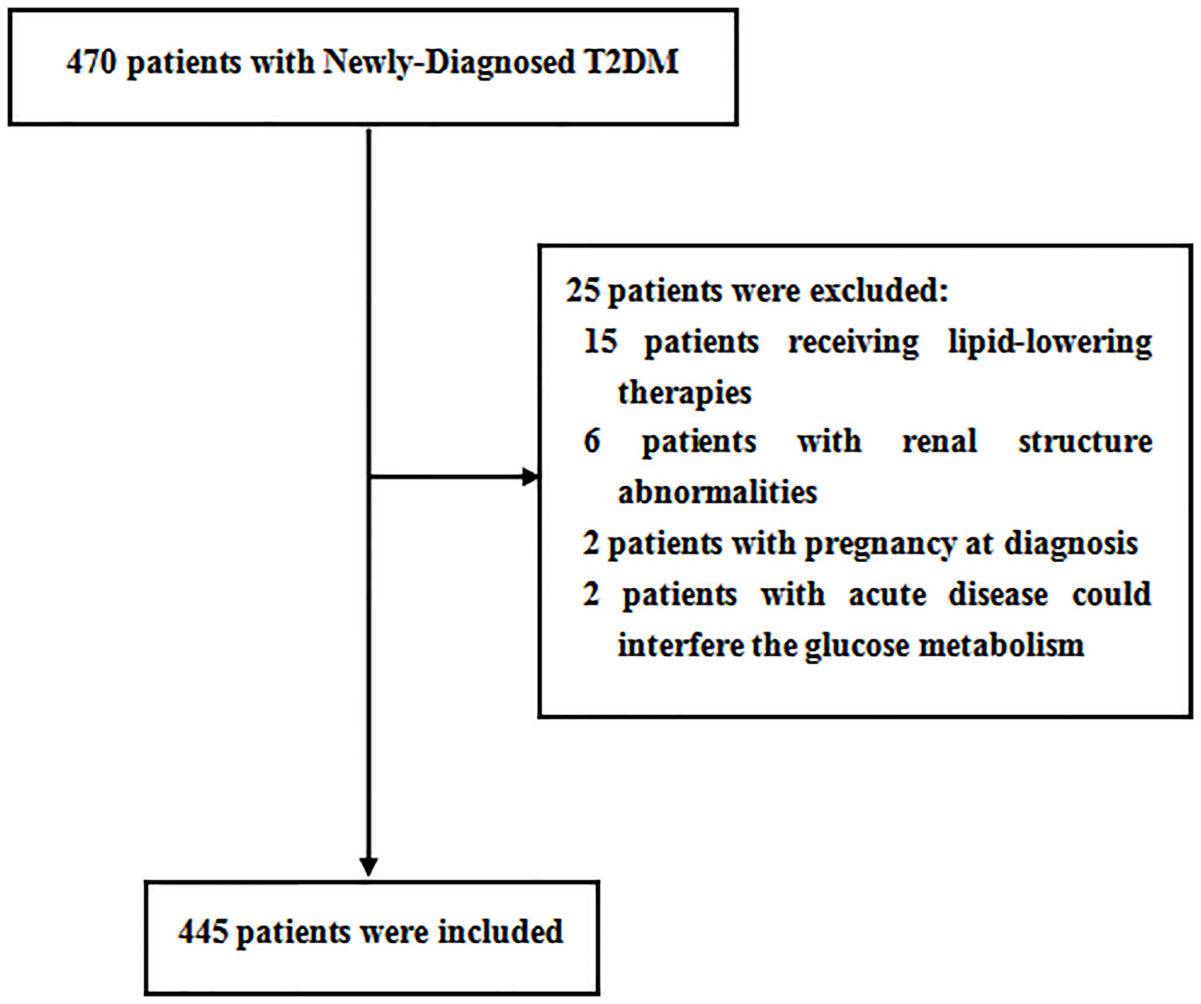
Flow diagram of the patients excluded and included in this study.

Overall, a total of 470 patients were screened; 445 patients meeting the inclusion and exclusion criteria were enrolled in this study. The flow diagram of the excluded and included patients is presented in **Figure 2**. Among the 445 patients, 226 (50.8%) patients were men. The mean age was 53.3 ± 7.9 years, ranging from 32 to 70 years old. The mean PrFT was 12.8 ± 4.8 mm. The prevalence of MetS was 57.5% (95% CI: 54.0–64.0%) in men and 58.9% (95% CI: 52.3–65.5%) in women separately. There was a significant difference in the mean PrFT between men and women (13.3 ± 5.1 *vs*. 12.2 ± 4.3 mm, *P <* 0.001). The characteristics of the patients divided into three groups based on tertiles of PrFT in men and women are shown in **Tables 1** and **2**. There was a significant difference in BMI, WC, TG, HDL-c, LDL-apolipoprotein B, UA, SBP, DBP, and HOMA-IR among groups both in men and women (*P* < 0.05). Patients in the higher-PrFT groups showed a higher level of BMI, WC, TG, UA, SBP, DBP, and HOMA-IR and a lower level of HDL-c compared with the lower-PrFT groups (*P* < 0.05). Moreover, patients in the higher-PrFT groups showed more patients that had MetS and hypertension (*P* < 0.05).

**Table 1:** The characteristics of newly diagnosed T2DM were divided into three groups based on tertiles of PrFT in men.

	Total	T1 (< 10.7 mm)	T2 (10.7–16.1 mm)	T3 (> 16.1 mm)	*P*
Age (year)	52.5 ± 8.2	52.4 ± 8.9	52.3 ± 7.7	52.8 ± 8.1	0.618
WC (cm)	86.6 ± 7.0	80.3 ± 3.3^a b^	86.9 ± 4.8^a c^	92.4 ± 6.4^b c^	< 0.001
BMI (kg/m^2^)	24.8 ± 3.1	22.0 ± 1.9^a b^	25.0 ± 1.9^a c^	27.2 ± 2.9^b c^	< 0.001
HbA1c (%)	8.8 ± 0.9	8.8 ± 1.0	8.7 ± 0.8	8.9 ± 0.9	0.327
TG (mmol/L)	2.3 ± 1.5	1.5 ± 1.0^a b^	2.0 ± 0.7^a c^	3.3 ± 1.9^b c^	< 0.001
TC (mmol/L)	5.4 ± 1.3	5.1 ± 1.1^a b^	5.5 ± 1.4^a^	5.6 ± 1.2^b^	0.026
HDL-c (mmol/L)	1.1 ± 0.2	1.3 ± 0.2^a b^	1.1 ± 0.1^a c^	0.9 ± 0.1^b c^	< 0.001
LDL-c (mmol/L)	3.6 ± 1.0	3.4 ± 0.9^a b^	3.8 ± 1.1^a^	3.7 ± 0.9^b^	0.014
APOA (g/L)	1.3 ± 0.3	1.4 ± 0.2^a b^	1.3 ± 0.2^a^	1.2 ± 0.3^b^	0.003
APOB (g/L)	1.1 ± 0.3	1.0 ± 0.3^a b^	1.1 ± 0.3^a^	1.1 ± 0.3^b^	0.018
UA (umol/L)	360.4 ± 86.2	307.5 ± 60.1^a b^	372.4 ± 77.2^a c^	401.4 ± 90.5^b c^	< 0.001
SBP (mmHg)	134.3 ± 18.4	119.1 ± 11.4^a b^	137.2 ± 10.2^a c^	146.1 ± 19.4^b c^	< 0.001
DBP (mmHg)	82.5 ± 10.3	76.6 ± 6.1^a b^	83.4 ± 12.2^a c^	87.5 ± 7.2^b c^	< 0.001
HOMA-IR	11.5 ± 6.5	6.3 ± 3.7^a b^	12.4 ± 4.7^a c^	15.6 ± 6.8^b c^	< 0.001
hs-CRP (mg/L)	3.4 ± 1.1	3.3 ± 0.9	3.4 ± 1.0	3.5 ± 0.9	0.218
Hypertension, *n* (%)	84 (37.2)	9 (12.0)^a b^	27 (36.5)^a c^	48 (62.3)^b c^	< 0.001
Smoking, *n* (%)	120 (53.1)	38 (50.7)	40 (54.1)	42 (54.5)	0.683
MetS, *n* (%)	130 (57.5)	11 (14.7)^a b^	24 (32.4)^a c^	69 (89.6)^b c^	< 0.001

BMI, body mass index; WC, waist circumference; HbA1c, glycated hemoglobin; UA, uric acid; TG, triglyceride; TC, total cholesterol; HDL-c, high-density lipoprotein cholesterol; LDL-c, low-density lipoprotein cholesterol; SBP, systolic blood pressure; DBP, diastolic blood pressure; HOMR-IR, Homeostasis Model Assessment—Insulin Resistance; hs-CRP, high-sensitivity C-reactive protein; MetS, metabolic syndrome.

a P < 0.05: T1 vs. T2.

b P < 0.05: T1 vs. T3.

c P < 0.05: T2 vs. T3.

**Table 2:** The characteristics of newly diagnosed T2DM were divided into three groups based on tertiles of PrFT in women.

	Total	T1 (< 10.2 mm)	T2 (10.2–14.9 mm)	T3 (> 14.9 mm)	*P*
Age (year)	54.2 ± 7.5	53.5 ± 7.7	54.1 ± 7.5	54.8 ± 7.3	0.569
WC (cm)	85.1 ± 6.7	80.4 ± 3.8^a b^	84.6 ± 5.0^a c^	90.2 ± 6.8^b c^	< 0.001
BMI (kg/m^2^)	24.2 ± 2.9	22.1 ± 2.1^a b^	24.1 ± 2.2^a c^	26.3 ± 2.6^b c^	< 0.001
HbA1c (%)	8.7 ± 1.0	8.7 ± 1.1	8.8 ± 0.9	8.7 ± 1.0	0.624
TG (mmol/L)	2.0 ± 1.1	1.3 ± 0.5^a b^	1.8 ± 0.6^a c^	2.9 ± 1.3^b c^	< 0.001
TC (mmol/L)	5.1 ± 1.1	4.9 ± 1.1^b^	5.2 ± 1.2	5.2 ± 1.0^b^	0.089
HDL-c (mmol/L)	1.1 ± 0.2	1.3 ± 0.2^a b^	1.1 ± 0.2^a c^	0.9 ± 0.2^b c^	< 0.001
LDL-c (mmol/L)	3.4 ± 0.9	3.1 ± 0.8^a b^	3.6 ± 0.9^a^	3.5 ± 1.0^b^	0.001
APOA (g/L)	1.3 ± 0.3	1.3 ± 0.3	1.3 ± 0.2	1.2 ± 0.3	0.519
APOB (g/L)	1.0 ± 0.3	0.9 ± 0.3^a b^	1.0 ± 0.3^a^	1.0 ± 0.3^b^	0.001
UA (umol/L)	348.9 ± 86.8	281.8 ± 62.3^a b^	352.5 ± 66.3^a c^	411.5 ± 76.8^b c^	< 0.001
SBP (mmHg)	132.3 ± 16.6	119.5 ± 12.2^a b^	132.6 ± 16.6^a c^	144.6 ± 9.7^b c^	< 0.001
DBP (mmHg)	81.0 ± 88.0	74.6 ± 5.7^a b^	82.0 ± 9.4^a c^	86.5 ± 6.5^b c^	< 0.001
HOMA-IR	10.7 ± 5.4	6.5 ± 3.7^a b^	11.4 ± 4.3^a c^	14.0 ± 5.1^b c^	< 0.001
hs-CRP (mg/L)	2.9 ± 0.8	3.0 ± 0.9	2.8 ± 1.2	2.8 ± 0.9	0.692
Hypertension, *n* (%)	81 (37.0)	11 (15.1)^a b^	24 (33.3)^a c^	46 (62.2)^b c^	< 0.001
Smoking, *n* (%)	6 (2.7)	3 (4.1)	3 (4.2)	0 (0)	0.207
MetS, *n* (%)	129 (58.9)	9 (12.3)^a b^	50 (69.4)^a c^	70 (94.6)^b c^	< 0.001

BMI, body mass index; WC, waist circumference; HbA1c, glycated hemoglobin; UA, uric acid; TG, triglyceride; TC, total cholesterol; HDL-c, high-density lipoprotein cholesterol; LDL-c, low-density lipoprotein cholesterol; SBP, systolic blood pressure; DBP, diastolic blood pressure; HOMR-IR, Homeostasis Model Assessment—Insulin Resistance; hs-CRP, high-sensitivity C-reactive protein; MetS, metabolic syndrome.

a P < 0.05: T1 vs. T2.

b P < 0.05: T1 vs. T3.

c P < 0.05: T2 vs. T3.

The main correlations between metabolic parameters and PrFT in the subgroup divided by sex are presented in **Table 3**. The results showed that PrFT was significantly and positively correlated with WC, BMI, TG, LDL-c, UA, SBP, DBP, and HOMA-IR in men and women groups. Moreover, PrFT was significantly and negatively correlated with HDL-c in both groups.

**Table 3:** Main correlations between metabolic parameters and PrFT in newly diagnosed T2DM divided by sex.

Parameter	Men (*n* = 226)		Women (*n* = 219)	
	*R*	*P*	*R*	*P*
WC (cm)	0.770	< 0.001	0.674	< 0.001
BMI (kg/m^2^)	0.779	< 0.001	0.690	< 0.001
HbA1c (%)	0.018	0.798	0.115	0.084
TG (mmol/L)	0.602	< 0.001	0.726	< 0.001
TC (mmol/L)	0.128	0.068	0.131	0.051
LDL-c (mmol/L)	0.156	0.019	0.190	0.005
HDL-c (mmol/L)	-0.592	< 0.001	-0.507	< 0.001
APOA (g/L)	-0.055	0.417	-0.046	0.528
APOB (g/L)	0.078	0.246	0.122	0.066
UA (umol/L)	0.494	< 0.001	0.665	< 0.001
SBP (mmHg)	0.695	< 0.001	0.713	< 0.001
DBP (mmHg)	0.538	< 0.001	0.611	< 0.001
hs-CRP (mg/L)	-0.067	0.364	-0.086	0.204
HOMA-IR	0.688	< 0.001	0.656	< 0.001

BMI, body mass index; HbA1c, glycated hemoglobin; WC, waist circumference; TG, triglyceride; TC, total cholesterol; HDL-c, high-density lipoprotein cholesterol; LDL-c, low-density lipoprotein cholesterol; UA, uric acid; SBP, systolic blood pressure; DBP, diastolic blood pressure; hs-CRP, high-sensitivity C-reactive protein; HOMR-IR: Homeostasis Model Assessment—Insulin Resistance.

The association between MetS and PrFT was further investigated by binomial logistic regression analysis divided by sex (**Table 4**). The PrFT was shown to be independently associated with MetS after adjustment for age (model 1). The ORs (95% CI) were 1.53 (1.38–1.70) in men and 1.66 (1.47–1.88) in women. After further adjustment for BMI, TC, LDL-c, UA, and HOMA-IR (model 2), the PrFT was shown to be independently associated with MetS. The ORs (95% CI) were 1.33 (1.16–1.53) in men and 1.50 (1.27–1.78) in women. After further additional adjustment for TG, WC, HDL-c, SBP, and DBP (model 3), the ORs remained significant. The ORs (95% CI) were 1.15 (1.03–1.38) in men and 1.31 (1.08–1.96) in women (*P* < 0.05).

**Table 4:** Binomial logistic regression analysis adjusted odds ratios (95% CIs) of PrFT in newly diagnosed T2DM divided by sex.

Parameter	Men (*n* = 226)		Women (*n* = 219)	
	OR (95% CI)	*P*	OR (95% CI)	*P*
Model 1	1.53 (1.38–1.70)	< 0.001	1.66 (1.47–1.88)	< 0.001
Model 2	1.33 (1.16–1.53)	< 0.001	1.50 (1.27–1.78)	< 0.001
Model 3	1.15 (1.03–1.38)	0.046	1.31 (1.08–1.96)	0.034

Model 1 was adjusted for age. Model 2 was adjusted for BMI, TC, LDL-C, UA, and HOMA-IR. Model 3 was additionally adjusted for TG, WC, HDL-c, SBP, and DBP.

BMI, body mass index; WC, waist circumference; HbA1c, glycated hemoglobin; UA, uric acid; TG, triglyceride; TC, total cholesterol; HDL-c, high-density lipoprotein cholesterol; LDL-c, low-density lipoprotein cholesterol; SBP, systolic blood pressure; DBP, diastolic blood pressure; HOMR-IR, Homeostasis Model Assessment Insulin Resistance.

**Figure 3:**
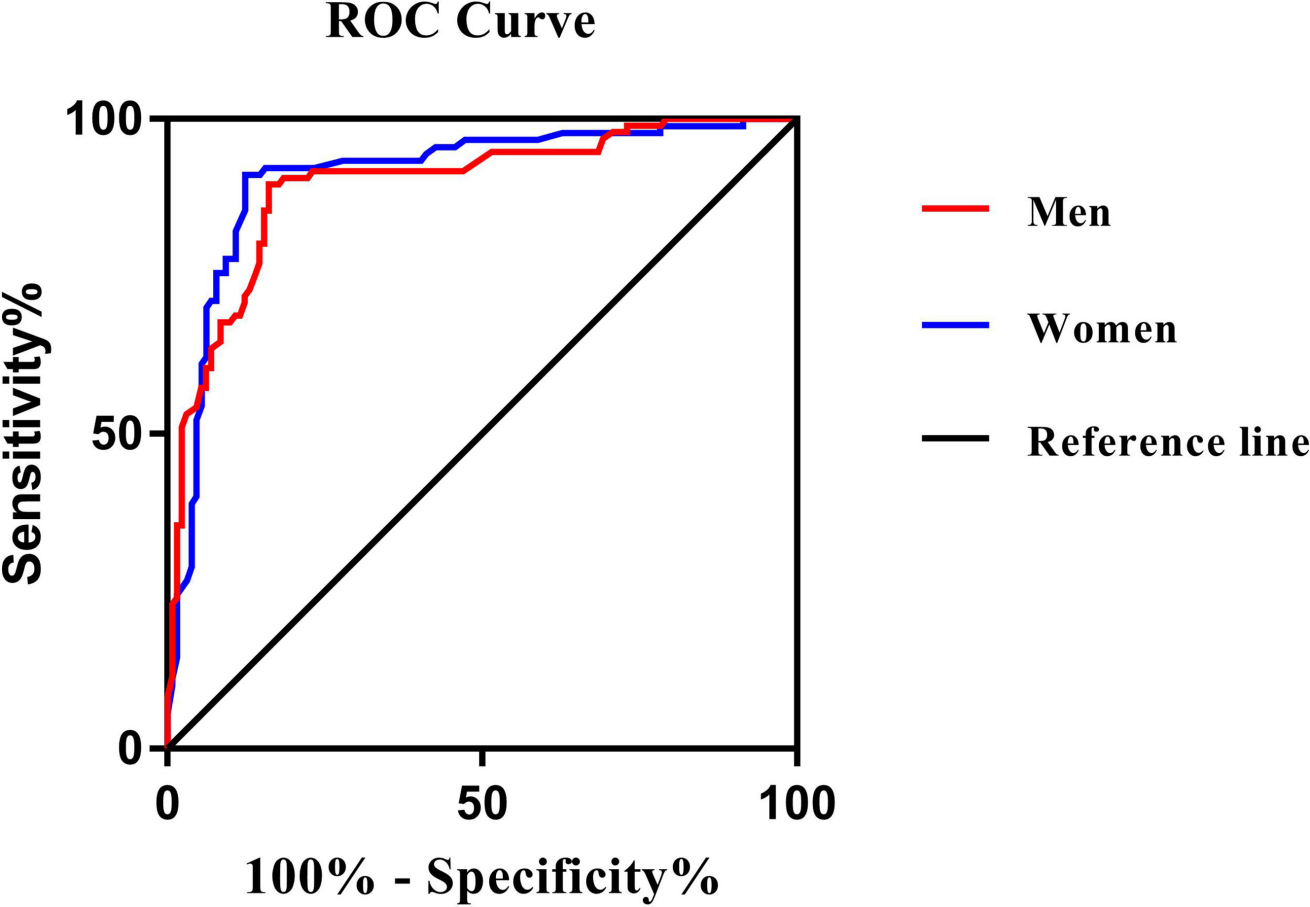
Receiver operating characteristic curves for the cutoff value of PrFT to identify MetS. In men, the area under the curve was 0.895 (95% CI: 0.852–0.939). In women, the area under the curve was 0.910 (95% CI: 0.876–0.953). The optimal cutoff values of PrFT were 14.6 mm (sensitivity: 83.8%, specificity: 89.6%) for men and 13.1 mm (sensitivity: 87.6%, specificity: 91.1%) for women.

The ROC curve analysis was used to further evaluate the ability of PrFT in identifying MetS divided by sex. From the ROC curve analysis, the results showed a good predictive value of PrFT for MetS. The areas under the curve of PrFT in identifying MetS were 0.895 (95% CI: 0.852–0.939, *P* < 0.001) in men and 0.910 (95% CI: 0.876–0.953, *P* < 0.001) in women (**Figure 3**). The optimal cutoff values of PrFT were 14.6 mm (sensitivity: 83.8%, specificity: 89.6%) for men and 13.1 mm (sensitivity: 87.6%, specificity: 91.1%) for women (**Table 5**).

**Table 5:** Receiver operating characteristic curve analysis of PrFT in identifying MetS in newly diagnosed T2DM divided by sex.

AUC (95% CI)	Cut-off value	Sensitivity (%) (95% CI)	Specificity (%) (95% CI)	PPV (%) (95% CI)	NPV (%) (95% CI)
Men (*n* = 226)					
0.895 (0.852–0.939)	14.6	83.8 (79.3–88.9)	89.6 (85.3–92.4)	91.6 (86.5–96.7)	80.4 (72.7–88.0)
Women (*n* = 219)					
0.910 (0.876–0.953)	13.1	87.6 (83.3–92.1)	91.1 (87.0–94.7)	93.4 (88.9–97.9)	83.7 (76.2–91.1)

MetS, metabolic syndrome; PrFT, perirenal fat thickness; PPV, positive predictive value; NPV, negative predictive value.

## Discussion

Diabetes is a metabolic disease characterized by chronic hyperglycemia that is often accompanied with MetS at the first diagnosis. MetS is a cluster of conditions that can increase the risk of cardiovascular diseases, heart disease, and stroke, which may increase all-cause mortality. Due to the complexity of MetS diagnosis, leading clinical practice often overlooked it. In the present study, the results confirmed that PrFT shows a close correlation with metabolic risk factors. Moreover, PrFT was significantly associated with higher odds of MetS after adjustment for other confounders. The ROC curves also showed a good predictive value of PrFT for MetS. The optimal cutoff values of PrFT in identifying MetS for Chinese newly diagnosed T2DM was 14.6 mm for men and 13.1 mm for women. It indicated that PrFT can be a surrogate marker for MetS in Chinese newly diagnosed T2DM.

Visceral fat and subcutaneous fat are the most important and common categories in adipose biology based on the anatomical and physiological characteristics of fat depots. Clinical evidences demonstrated that Asians are more likely to have more obesity-related consequences in patients with lower WC and BMI due to more visceral fat mass deposition compared with Caucasians (18). CT scan is a reliable tool to quantify adipose tissue depots. The density of adipose tissue in Hounsfield unit (HU) can be used to distinguish visceral fat from other tissues. Among visceral adipose tissue deposits, perirenal fat located in the retroperitoneal space and surrounding the kidneys can be quantitatively measured by radiological diagnosis for renal positioning, and the posterolateral perirenal fat thickness measured by CT scanning had shown a positive correlation with perirenal fat mass (8). The anatomical structure and location of perirenal fat determined its specific biological characteristics. Compared with other adipose tissues classified as loose connective tissues, perirenal fat has a complete system of blood supply, lymph fluid drainage, innervation, and other special morphological features, which make it similar to other internal organs and different from traditionally classified connective tissues (9). These special anatomical structure ensured that perirenal fat can modulate the metabolic system through neural reflexes (19), adipokine secretion (20), adipocyte interactions (21), and paracrine substance (22). Among them, adipokines (leptin, adiponectin, apelin, and nesfatin) play important regulatory roles in endocrine metabolic systems, insulin sensitivity, and lipolysis *via* the autocrine, paracrine, and endocrine pathways (23, 24). In addition, other bioactive factors, such as leptin, adiponectin, tumor necrosis factor-α, interleukin-6, interleukin-8, and MCP-1, can also be released from perirenal fat, which is involved in the pathogenesis of CVD, metabolic disorders, and T2DM (25, 26). Thus, these specific biological and anatomical characteristics provided a basis for the involvement of perirenal fat in MetS regulation.

Clinical studies have also observed the association between PrFT and metabolic risk factors. A study that enrolled overweight and obese subjects showed that PrFT was independently associated with HDL-c and WC (13). Another study has also shown that PrFT was significantly correlated with metabolic risk factors such as UA, TG, and WC in patients with chronic kidney disease (27). Moreover, PrFT also showed a positive independent association between PrFT and mean 24-h diastolic blood pressure levels in overweight and obese subjects (28). The results in our study also showed a positive correlation between PrFT and HOMA-IR, which was confirmed to participate in the occurrence and development of MetS and T2DM (29). Meanwhile, PrFT is also reported to be associated with other metabolic diseases and T2DM complications. Satsuki K et al. have also demonstrated that PrFT can be a reliable method for the quantification of fatty liver as well as for the quantification of visceral fat (30). Increasing evidence have suggested that the accumulation of perirenal fat increases the risk for the development of chronic kidney disease through decreasing the eGFR level and increasing the excretion rate of urinary protein (31–33). The results in our study were consistent with these previous studies. PrFT was correlated with metabolic risk factors like WC, TG, HDL-c, SBP, DBP, UA, and HOMA-IR. As expected, PrFT was significantly independent with higher odds (95% CI) of MetS after adjustment for other confounders. The ROC curve analysis results in our study showed a good predictive value of PrFT for MetS both in men and women, which indicated that PrFT can be a surrogate marker for MetS in newly diagnosed T2DM.

To our knowledge, this is the first study to have confirmed the predictive value of PrFT for MetS in Chinese newly diagnosed T2DM. There are some limitations in our study. Firstly, due to the fact that the prevalence of MetS may vary in geographic distribution and race (34), the optimal cutoff values of PrFT may not be applicable to other races. Secondly, although CT scanning can accurately measure the PrFT, the radiation may limit its use in clinical practice. In conclusion, in this cross-sectional study, a surrogate marker for MetS in Chinese newly diagnosed T2DM was found. PrFT was significantly independent with MetS and showed a powerful predictive value for MetS, which suggested that PrFT can be a surrogate marker for MetS in Chinese newly diagnosed T2DM.

## Data Availability Statement

The original contributions presented in the study are included in the article/supplementary material. Further inquiries can be directed to the corresponding author.

## Ethics Statement

The studies involving human participants were reviewed and approved by the Ethical Committee of Longyan First Affiliated Hospital of Fujian Medical University. The patients/participants provided their written informed consent to participate in this study.

## Author Contributions

WW contributed to data curation and writing—review and editing. WW, XG, YC and MT conducted the investigation. XG took charge of the software and contributed to writing—original draft. All authors contributed to the article and approved the submitted version.

## Conflict of Interest

The authors declare that the research was conducted in the absence of any commercial or financial relationships that could be construed as a potential conflict of interest.

## Publisher’s Note

All claims expressed in this article are solely those of the authors and do not necessarily represent those of their affiliated organizations, or those of the publisher, the editors and the reviewers. Any product that may be evaluated in this article, or claim that may be made by its manufacturer, is not guaranteed or endorsed by the publisher.
